# Utility of Tokyo Guidelines 2018 in early laparoscopic cholecystectomy for mild and moderate acute calculus cholecystitis: A retrospective cohort study

**DOI:** 10.3389/fsurg.2022.1022258

**Published:** 2023-01-16

**Authors:** Yong Yan, Yinggang Hua, Wei Yuan, Xuanjin Zhu, Yongliang Du, Shanfei Zhu, Bailin Wang

**Affiliations:** Department of General Surgery, Guangzhou Red Cross Hospital, Jinan University, Guangzhou, China

**Keywords:** Tokyo Guidelines 2018, laparoscopic cholecystectomy, acute calculus cholecystitis, subtotal cholecystectomy, retrospective cohort study

## Abstract

**Background:**

Tokyo Guidelines 2018 (TG18) proposed laparoscopic cholecystectomy (LC) for acute calculus cholecystitis (ACC) irrespective of the duration of symptoms. This retrospective study assessed the impact of utility of TG18 in early LC for ACC.

**Methods:**

From 2018 to 2020, 66 patients with mild (grade I) and moderate (grade II) ACC who underwent early surgery were studied. Subgroup analyses were based on timing of surgery and operation time.

**Results:**

A total of 32 and 34 patients were operated within and beyond 7 days since ACC onset. More patients with grade II ACC were in the beyond 7 days group (*P *< 0.05). More patients with enlarged gallbladder were in the within 7 days group (*P *< 0.05). The duration of symptoms to admission, symptoms to LC, and operation time were longer in the beyond 7 days group (*P *< 0.05). There were no significant differences regarding intraoperative blood loss, conversion to bail-out procedures, complication rate, hospital stay, and cost between the two groups (*P *> 0.05). Longer operation time was significantly associated with duration of symptoms to admission, symptoms to LC, and conversion to laparoscopic subtotal cholecystectomy (LSC) (*P *< 0.05).

**Conclusion:**

In a subset of carefully selected patients, applying TG18 in early LC for mild and moderate ACC results in acceptable clinical outcomes. Standardized safe steps and conversion to LSC in difficult cases are important.

## Introduction

Acute calculus cholecystitis (ACC) is a very common inflammatory disease of the gallbladder and the most common complication of gallstone disease (1). In recent decades, several guidelines, including the Tokyo Guidelines (TG) and World Society of Emergency Surgery (WSES) guidelines, have been established to optimize the management of ACC (2, 3). For patients with ACC, these guidelines recommend laparoscopic cholecystectomy (LC) as the first-line treatment. Despite the high frequency of ACC and relevant guidelines in practice, significant controversies remain regarding the diagnosis and management of ACC. With regard to the treatment of ACC, the main controversies were around the timing of surgery.

In TG07 and TG13, management of ACC was recommended according to the time since symptoms onset and grading of ACC severity (4). WSES guidelines recommend early LC be performed as soon as possible within 7 days from hospital admission and within 10 days from the onset of symptoms. In the recent updated TG18, early LC was proposed for ACC if a patient is deemed capable of withstanding surgery, and advanced laparoscopic techniques are available, regardless of exactly how much time has passed since symptoms onset and the grading of cholecystitis severity (5). Ours is a tertiary referral center for medical and surgical specialties. We diagnose and manage of ACC in accordance with the TG18, where mild (Grade I) and moderate (Grade II) ACC were proposed to early LC irrespective of time since symptoms onset. The aim of this study was to compare the clinical outcomes of patients who underwent early LC for mild and moderate ACC according to the timing of surgery in a cohort of patients. Based on our findings, the feasibility and impact of the utility of TG18 in early LC for mild and moderate ACC were assessed.

## Methods

**Figure 1:**
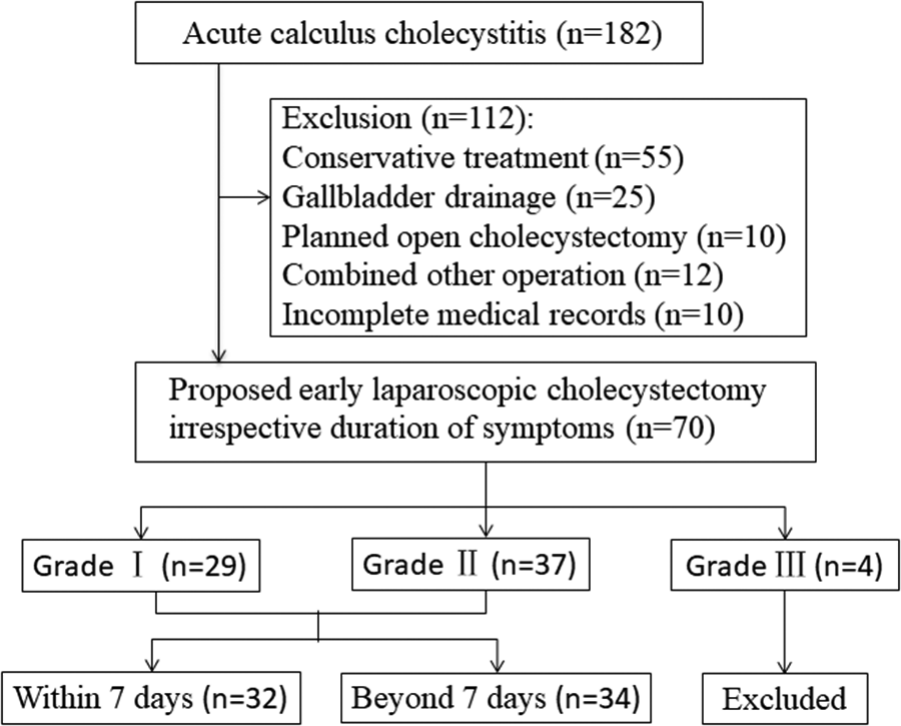
Study design and patient flow across the study.

This retrospective study was conducted at the Guangzhou Red Cross Hospital, and more than 200 LCs are performed annually. All patients with grade I and grade II ACC who underwent early surgery from 1 January 2018 to 31 December 2020 were included (Figure 1). Exclusion criteria were as follows: (i) previous biliary surgery history, (ii) intrahepatic biliary or common bile duct stones, (iii) malignant pancreatic or biliary tumors, (iv) suppurative cholangitis or biliary pancreatitis, (v) intended open or delayed cholecystectomy after primary admission or percutaneous cholecystostomy, (vi) American Society of Anesthesiologists (ASA) score ≥ 4, (vii) without complete medical records, and (viii) pregnancy. This study was approved by the Ethics Committee of the Guangzhou Red Cross Hospital (2019-099-01). Because of the retrospective study design, written informed consent was not obtained from the patients.

Patient data were retrospectively extracted from medical records as follows: age, sex, body mass index (BMI), preoperative comorbidities, ASA score, grade of ACC, preoperative laboratory and image findings, operation time, intraoperative blood loss, conversion to bail-out procedures, early surgical complications, postoperative oral intake, length of hospital stay, cost, and 30-day mortality. Early surgical complications were considered when occurring within 30 days after surgery, including bile duct injury, wound infection, bile leakage, postoperative bleeding, choledocholithiasis, abdominal collection, and ileus.

### Diagnosis of ACC and indication for LC

Following TG18, diagnosis of ACC was assessed by three criteria: (1) local signs of inflammation, such as Murphy's sign, or right upper quadrant mass/pain/tenderness; (2) systemic signs of inflammation, such as fever, elevated C-reactive protein (CRP), or elevated white blood cell (WBC); (3) imaging findings (6). The generally accepted imaging findings of ACC are thickening of the gallbladder wall (≥4 mm), enlargement of the gallbladder (long axis ≥ 8 cm, short axis ≥ 4 cm), gallstones or retained debris, fluid accumulation around the gallbladder, and linear shadows in the fatty tissue around the gallbladder (7). A preoperative imaging finding of ACC was determined based on abdominal ultrasonography, abdominal CT, or magnetic resonance cholangiopancreatography (MRCP).

When ACC is diagnosed, the severity is determined and initial treatment includes monitoring of respiration and hemodynamics, sufficient intravenous fluid, electrolyte infusion and electrolyte correction, as well as antimicrobials and analgesics (8). All patients were evaluated for surgical risk using the Charlson comorbidity index (CCI) and ASA score. In principle, early LC is proposed for the cases of Grade I and II with CCI 5 or less and/or ASA score II or less. However, in patients with surgical risk in admission, general supportive care is offered first and then assessment of severity and surgical risk is repeated every 24 h. After improvement with initial medical treatment, the Grade I and II cases with CCI 5 or less and/or ASA score II or less could indicate LC.

### Surgical procedure

**Figure 2:**
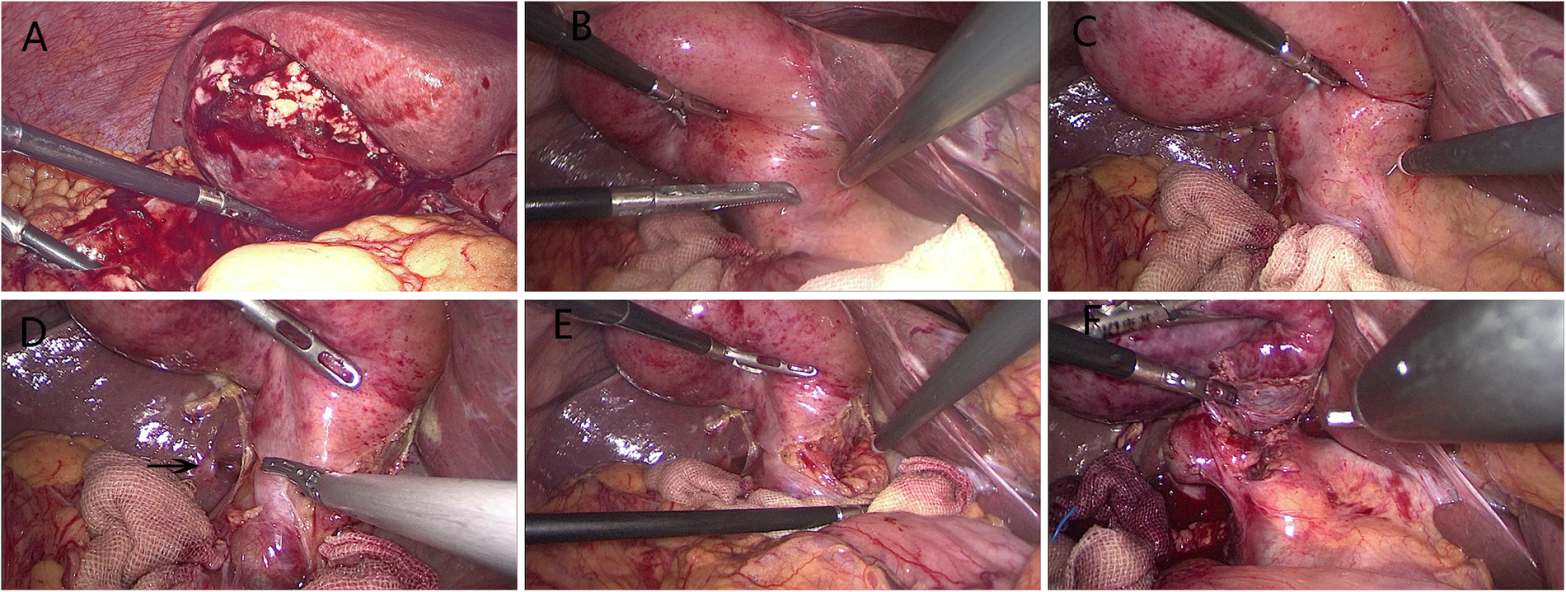
Laparoscopic cholecystectomy for ACC. (**A**) Typical finding of grade II ACC with empyema and gangrene. (**B**) Typical finding of grade I ACC with hyperemia and edema. (**C**) Effective retraction of the gallbladder to develop a plane in Calot's triangle area. (**D**) Dissection from the posterior leaf of the peritoneum covering the neck of the gallbladder and above Rouviere's sulcus (arrow). (**E**) Maintaining the plane of dissection on the gallbladder surface. (**F**) Dissecting the lower part of the gallbladder bed to obtain the critical view of safety.

Patients were placed in a reverse Trendelenburg position under general anesthesia. We tended to use a three-trocar method: the first 10 mm trocar was placed in the subumbilical area for carbon dioxide insufflation and laparoscope. The second 10 mm trocar was located in the area below the xiphoid. The third 5 mm trocar was placed in the midclavicular line, 1–2 cm under the right costal margin. Sometimes, an additional 5 mm trocar in the right anterior axillary line was needed when the inflammation or adhesion was severe in the operation area. Based on TG18, we adopted the standardized safe steps in LC that included decompression of tense gallbladder, appropriate retraction of the gallbladder to develop a plane in Calot's triangle area, exposing the gallbladder surface above Rouviere's sulcus, maintaining the plane of dissection on the gallbladder surface, dissecting at least one-third lower part of the gallbladder bed, and creation of the critical view of safety (CVS) (Figure 2) (9). An attempt to dissect at the area of Calot's triangle was made in all cases. Dissection was completed using electrocautery hook dissector, scissors, or ultrasonic shears. We routinely freed the cystic artery and cystic duct, achieved a CVS, and then the cystic duct and artery were sealed and dissected with Hemo-lock. After gallbladder dissection, securing hemostasis, intra-abdominal cavity irrigation, leaving a drain in the subhepatic space, and removal of the collecting bag through the 10 mm umbilical port was done.

**Figure 3:**
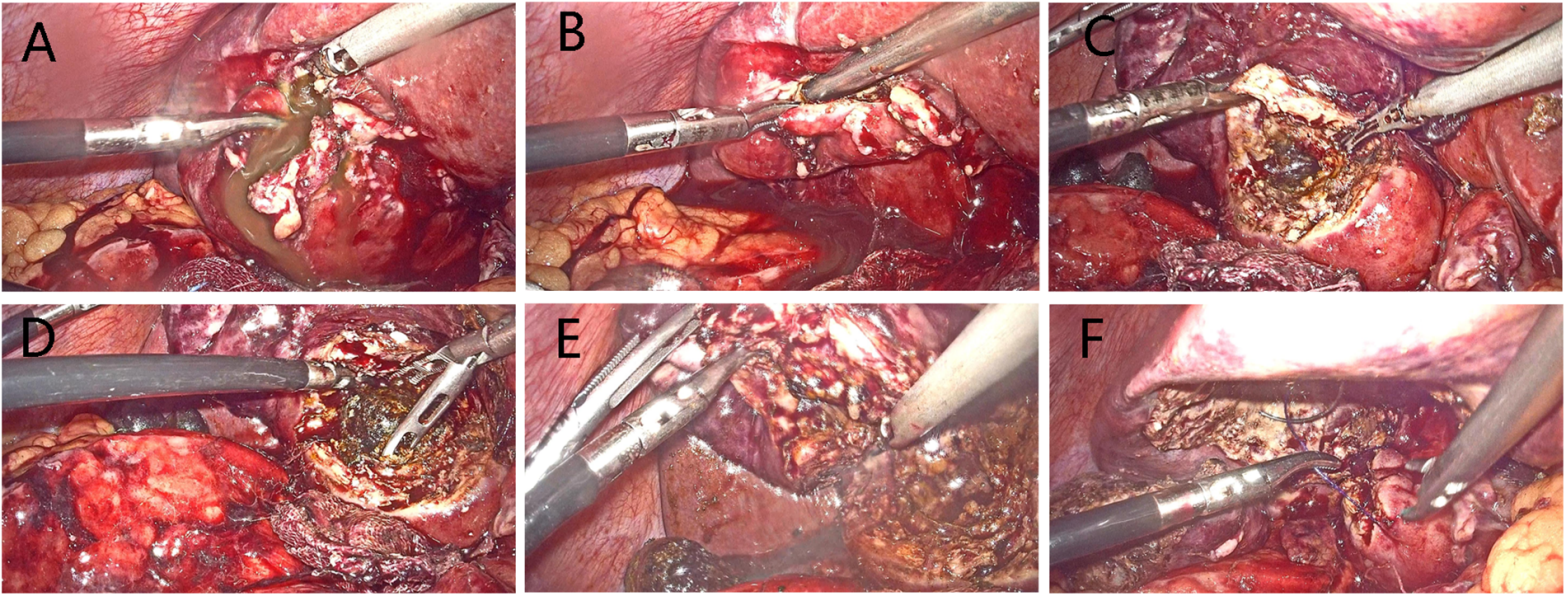
Procedures of laparoscopic subtotal cholecystectomy. (**A**) Making an incision in the gallbladder fundus. (**B**) Aspirating the contents to decompress gallbladder. (**C**) Opening of Hartmann's pouch. (**D**) Clearing the stones obstructing the gallbladder outlet. (**E**) Removing as much of the gallbladder wall as possible. (**F**) Suture of gallbladder remnant.

The fundus first technique was attempted in some cases in which the cystic duct and common bile duct were difficult to be identified (10). If a CVS showing anatomically important landmarks cannot be achieved, the surgery was converted to a bail-out procedure, open approach, or laparoscopic subtotal cholecystectomy (LSC), as it is introduced in TG18. LSC included opening of Hartmann's pouch, aspiration of bile, removal of all gallstones, removing as much of the gallbladder wall as possible, and then closed gallbladder remnant using barbed sutures (Figure 3) (11).

### Postoperative management

Antibiotics were administered once immediately the diagnosis of ACC was confirmed; postoperative antimicrobial therapy was continued until the patient is afebrile, with generally normal WBC, and without abdominal signs of infection. The patients were encouraged to be up and about at postoperative 1 day, and liquid food intake could begin 24 h after surgery for patients with resuming peristaltic sound of gut. The abdominal cavity drainage tube was removed at postoperative 1 day if without abnormal drainage. Abdominal signs, complete blood count, liver function, serum amylase, and abdominal ultrasonography were monitored. MRCP or CT was performed if clinically indicated. Discharge criteria were normal inflammatory markers, no special discomfort, and no abnormal image finding.

### Statistical analysis

Patients’ characteristics were summarized by mean ± standard deviation (SD) for continuous variables with approximately normal distribution or median and interquartile range for those with skewed distributions. Continuous variables were compared between groups using two-sided independent two-sample *t* tests or the Wilcoxon rank-sum tests depending on normality. Categorical variables were expressed as absolute numbers and percentages, and compared using the Chi-square test or Fisher's exact test. The relationship between timing of LC and operation time was analyzed with a receiver operating characteristic (ROC) curve and area under curve (AUC). The cut-off value was determined using Youden's index. Univariate analyses were performed to investigate influential factors for operation time. A probability value of <0.05 was considered to indicate statistical significance. Data analysis was performed using SPSS 20.0 (IBM, United States).

## Results

A total of 182 patients diagnosed with ACC between January 2018 and December 2020 were included. After exclusion of treatment other than LC and incomplete records, 70 patients had undergone early LC at the same hospital as they presented to our center irrespective of the duration of symptoms. Of these, four patients categorized as severe (Grade III) ACC also underwent LC after assessment and improvement of initial medical treatment 24 h later. So excluding 4 Grade III ACC patients, a total of 66 patients with mild (Grade I) and moderate (Grade II) ACC were included in this study, of which, 50 patients had a CT scan to confirm the diagnosis of ACC (included 10 patients had combined ultrasonic diagnosis), 16 patients had only ultrasonic diagnosis, and 6 patients had undergone additional MRCP.

### Demographic details and operative outcomes of included patients

The preoperative and operative data of the study population are shown in Tables 1, 2. There were 33 males and 33 females, with a mean age of 61.8 years and mean BMI of 22.8 kg/m^2^. Thirty-two (48.5%) patients had hypertension, 10 (15.2%) had diabetes mellitus, 7 (10.6%) had coronary heart disease, 3 (4.5%) had chronic obstructive pulmonary disease, 4 (6.1%) had cerebral diseases, and 2 (3.0%) had chronic renal failure. Sixty (90.9%) patients had an ASA score of I or II and 6 (9.1%) had ASA score of III; 29 (43.9%) had mild (grade I) ACC and 37 (56.1%) had moderate (grade II) ACC. Twenty-three (34.8%) patients manifested with recurrent cholecystitis as their previous episode of ACC, and 3 (4.5%) had a history of previous abdominal surgery. The median duration of symptoms to admission, admission to LC, and symptoms to LC were 3, 3, and 7.5 days, respectively.

**Table 1:** Demographic and preoperative data of patients included in the study.

Parameter	Total (*n* = 66)	LC within 7 days (*n* = 32)	LC beyond 7 days (*n* = 34)	*P-*value
Age (years)	61.8 ± 14.3	59.5 ± 13.1	63.9 ± 15.3	0.211
Gender (female/male)	33/33	16/16	17/17	1.000
BMI (kg/m^2^)	22.8 ± 2.1	23.2 ± 2.3	22.5 ± 1.9	0.231
Comorbidity, *n* (%)				
Hypertension	32 (48.5)	17 (53.1)	15 (44.1)	0.464
Diabetes mellitus	10 (15.2)	3 (9.4)	7 (20.6)	0.306
Coronary heart disease	7 (10.6)	3 (9.4)	4 (11.8)	1.000
COPD	3 (4.5)	2 (6.2)	1 (2.9)	0.608
Cerebral diseases	4 (6.1)	3 (9.4)	1 (2.9)	0.348
Chronic renal failure	2 (3.0)	1 (3.1)	1 (2.9)	1.000
ASA score, *n* (%)				
I, II	60 (90.9)	29 (90.6)	31 (91.2)	1.000
III	6 (9.1)	3 (9.4)	3 (8.8)	
Grade of ACC, *n* (%)				
Mild	29 (43.9)	19 (59.4)	10 (29.4)	0.014
Moderate	37 (56.1)	13 (40.6)	24 (70.6)	
Recurrent ACC	23 (34.8)	10 (31.2)	13 (38.2)	0.552
Previous abdominal surgery, *n* (%)	3 (4.5)	2 (6.2)	1 (2.9)	0.608
Symptoms, *n* (%)				
Abdominal pain/distension	66 (100)	32 (100)	34 (100)	1.000
Fever	16 (24.2)	10 (31.2)	6 (17.6)	0.197
Nausea/vomiting	29 (43.9)	15 (46.9)	14 (41.2)	0.641
Jaundice	3 (4.5)	2 (6.2)	1 (2.9)	0.608
Duration of symptoms to admission (days)	3.0 (1.0–6.0)	1.0 (1.0–3.0)	5.5 (4.7–7.0)	0.001
Duration of admission to LC (days)	3.0 (2.0–4.0)	3.0 (1.0–4.0)	3.0 (2.0–5.0)	0.212
Duration of symptoms to LC (days)	7.5 (5.0–9.0)	5.0 (3.2–6.0)	9.0 (8.0–9.0)	0.001
Preoperative blood examination				
WBC (10^9^/L)	12.6 ± 4.5	13.0 ± 4.4	12.3 ± 4.7	0.497
CRP	37.8 (16.3–115.8)	28.1 (14.1–111.3)	40.4 (16.8–141.8)	0.419
Alkaline phosphatase (U/L)	83.5 (64.0–127.0)	79.3 (68.0–116.7)	86.5 (56.7–144.2)	0.715
Alanine aminotransferase (U/L)	20.5 (12.0–50.0)	17.5 (11.0–34.7)	23.0 (13.0–59.2)	0.253
Aspartate aminotransferase (U/L)	21.0 (16.7–38.5)	21.0 (16.0–37.7)	20.5 (17.0–40.5)	0.974
Total bilirubin (μmol/L)	14.1 (9.6–21.3)	14.4 (10.3–21.0)	14.0 (8.0–21.8)	0.832
Preoperative image findings				
Gallbladder thickening (≥4 mm), *n* (%)	28 (42.4)	11 (34.4)	17 (50.0)	0.199
Enlargement of gallbladder, *n* (%)	37 (56.1)	24 (75.0)	13 (38.2)	0.003
Gallstones in gallbladder neck/duct, *n* (%)	22 (33.3)	11 (34.4)	11 (32.4)	0.862
Three or more gallstones, *n* (%)	42 (63.6)	19 (59.4)	23 (67.6)	0.485

LC, laparoscopic cholecystectomy; BMI, body mass index; ASA, American Society of Anesthesiologists; ACC, acute calculus cholecystitis; COPD, chronic obstructive pulmonary disease; WBC, White blood cell; CRP, C-reactive protein.

**Table 2:** Comparison of operative outcomes of LC between the within 7 days and beyond 7 days groups.

Parameter	Total (*n* = 66)	LC within 7 days (*n* = 32)	LC beyond 7 days (*n* = 34)	*P-*value
Operation time (min)	120.0 (100.0–150.0)	105.0 (90.0–126.0)	129.0 (120.0–160.0)	0.002
Intraoperative blood loss (ml)	20.0 (20.0–50.0)	20.0 (20.0–50.0)	20.0 (20.0–100.0)	0.339
Bail-out procedure, *n* (%)				
Open conversion	4 (6.1)	2 (6.2)	2 (5.9)	1.000
LSC	12 (18.2)	4 (12.5)	8 (23.5)	0.246
Surgical complications, *n* (%)				
Wound infection	5 (7.6)	2 (6.2)	3 (8.8)	1.000
Abdominal collection	7 (10.6)	4 (12.5)	3 (8.8)	0.705
Choledocholithiasis	3 (4.5)	1 (3.1)	2 (5.9)	1.000
Bile leak	3 (4.5)	2 (6.2)	1 (2.9)	0.608
Adhesive ileus	1 (1.5)	1 (3.1)	0 (0)	0.485
Postoperative bleeding	0	0	0	1.000
Bile duct injury	0	0	0	1.000
Time to oral intake (days)	2.0 (1.0–2.0)	2.0 (1.0–2.0)	1.5 (1.0–3.0)	0.841
Postoperative hospital stay (days)	7.0 (5.0–9.2)	7.0 (5.0–9.0)	7.0 (5.0–10.2)	0.851
Total hospital stay (days)	10 (8.0–13.0)	9.5 (8.0–13.0)	10.5 (8.7–13.2)	0.402
Medicine expenses (CNY)	5,253.6 (3,174.7–8,256.8)	4,496.5 (2,918.5–6,728.3)	5,544.1 (3,796.4–10,017.3)	0.144
Total expenses (CNY)	30,063.1 (25,278.4–40,026.5)	29,697.6 (24,439.3–34,033.7)	30,063.1 (27,699.9–43,866.7)	0.248
Mortality, *n* (%)	0	0	0	1.000

LC, laparoscopic cholecystectomy; LSC, laparoscopic subtotal cholecystectomy; CNY, China Yuan.

The median operation time was 120 min, and median intraoperative blood loss was 20 ml. LC was converted to a bail-out procedure of LSC and open cholecystectomy in 12 (18.2%) and 4 (6.1%) of the 66 patients, respectively. The most common reasons for conversion included severe inflammation, fibrosis or scarring of the gallbladder, nondissectable adhesions, and bleeding from Calot's triangle. The median time to oral intake was 2 days. The median of total and postoperative hospital stay was 10 and 7 days, respectively.

No major postoperative complications requiring any re-surgery occurred. A total of 19 early surgical complications occurred in 13 (19.7%) patients, which included wound infection (5), abdominal infection (7), choledocholithiasis (3), bile leakage (3), and adhesive ileus (1). There was no bile duct injury, postoperative bleeding, and 30-day mortality.

### Comparison between within and beyond 7 days groups

The comparisons of within vs. beyond 7 days group are shown in Tables 1, 2. Among them, 32 patients operated within 7 days and 34 operated beyond 7 days since symptoms’ onset. Comparisons stratified as per timing of surgery showed no obvious differences in terms of age, gender, BMI, comorbidity, ASA score, previous abdominal surgery, symptoms, duration of admission to LC, and preoperative blood examination (*P *> 0.05). There was no obvious difference of preoperative imaging regarding gallbladder thickening, gallstones’ location, and gallstones number between the two groups (*P *> 0.05). There were longer duration of symptoms to admission, symptoms to LC, and more patients with ACC of grade II in beyond 7 days group than in within 7 days group (*P *= 0.001, *P *= 0.001, and *P *= 0.014, respectively). However, there were more patients with an enlarged gallbladder in the within 7 days group than in the beyond 7 days group (*P *= 0.003).

The operation time in the beyond 7 days group was longer than that in the within 7 days group (*P *= 0.002). There were no significant differences regarding intraoperative blood loss, conversion to bail-out procedures, time to oral intake, postoperative and total hospital stay, medicine expenses, and total expenses between the two groups (*P *> 0.05).

The complication rate showed no significant difference between the two groups (*P *> 0.05). Bile leakage was present in two patients of the within 7 days group and one patient of the beyond 7 days group; all were settled by adequate drainage and use of antibiotics. Abdominal collection happened in four patients of the within 7 days group and three patients of the beyond 7 days group, which was managed with anti-infection treatment and percutaneous drainage. Three patients developed abdominal pain and jaundice after LC and routine abdominal CT revealed choledocholithiasis; one from the within 7 days group was resolved by endoscopic retrograde cholangiopancreatography (ERCP) and endoscopic sphincterotomy, two from the beyond 7 days group had spontaneous normalization of clinical symptoms and laboratory values, and further MRCP showed no stones in the common bile duct and not requiring ERCP. Wound infection happened in five patients, two from the within 7 days group and three from the beyond 7 days group, and was managed successfully through wound dressing and local drainage. One patient in the within 7 days group developed adhesive ileus that was resolved by conservative treatment.

### Comparison of patient data according to operation time

The median operation time was 105 min in the within 7 days group and 129 min in the beyond 7 days group (*P *= 0.002). The ROC curve for the beyond 7 days group showed an AUC for operation time of 0.716 (*P *= 0.003), and the cut-off value for operation time was 104 min. Assuming a cut-off value of operation time of 104 min, it was <104 min in 21 patients and ≥104 min in 45 patients (Tables 3, 4). We analyzed the factors affecting the operation time, and univariate analyses showed that longer operation time was significantly associated with the duration of symptoms to admission (*P *= 0.001), symptoms to LC (*P *= 0.012), and conversion to LSC (*P *= 0.007).

**Table 3:** Comparison of preoperative data according to operation time.

Parameter	<104 min (*n* = 21)	≥104 min (*n* = 45)	*P-*value
Age (years)	60.9 ± 14.3	62.2 ± 14.5	0.735
Gender (female/male)	11/10	22/23	0.792
BMI (kg/m^2^)	22.8 ± 2.1	22.9 ± 2.1	0.879
Comorbidity, *n* (%)			
Hypertension	12 (57.1)	20 (44.4)	0.336
Diabetes mellitus	3 (14.3)	7 (15.6)	1.000
Coronary heart disease	0 (0)	7 (15.6)	0.087
COPD	1 (4.8)	2 (4.4)	1.000
Cerebral diseases	3 (14.3)	1 (2.2)	0.091
Chronic renal failure	0 (0)	2 (3.0)	1.000
ASA score, *n* (%)			
I, II	20 (95.2)	40 (88.9)	0.656
III	1 (4.8)	5 (11.1)	
Grade of ACC, *n* (%)			
Mild	12 (57.1)	17 (37.8)	0.140
Moderate	9 (42.9)	28 (62.2)	
Recurrent ACC	6 (28.6)	17 (37.8)	0.465
Previous abdominal surgery, *n* (%)	0 (0)	3 (6.7)	0.546
Symptoms, *n* (%)			
Abdominal pain/distension	21 (100)	45 (100)	1.000
Fever	3 (14.3)	13 (28.9)	0.197
Nausea/vomiting	11 (52.4)	18 (40.0)	0.345
Jaundice	1 (4.8)	2 (4.4)	1.000
Duration of symptoms to admission (days)	1 (1–2.5)	5 (3–6.5)	0.001
Duration of admission to LC (days)	3.0 (2.0–5.0)	2.0 (2.0–4.0)	0.106
Duration of symptoms to LC (days)	5.0 (3.5–7.0)	8.0 (6.0–9.0)	0.012
Preoperative blood examination			
WBC (10^9^/L)	13.5 ± 4.1	12.2 ± 4.7	0.296
CRP	25.0 (10.8–110)	41.1 (17.6–116.7)	0.321
Alkaline phosphatase (U/L)	75.0 (68.0–98.5)	106.0 (62.0–150.5)	0.130
Alanine aminotransferase (U/L)	28.0 (15.5–147.0)	20.0 (11.5–43.5)	0.181
Aspartate aminotransferase (U/L)	25.0 (16.0–43.5)	20.0 (17.0–33.5)	0.644
Total bilirubin (μmol/L)	14.9 (10.4–24.5)	14.0 (9.0–20.4)	0.540
Preoperative image findings			
Gallbladder thickening (≥4 mm), *n* (%)	9 (42.9)	19 (42.2)	0.961
Enlargement of gallbladder, *n* (%)	14 (66.7)	23 (51.1)	0.236
Gallstones in gallbladder neck/duct, *n* (%)	6 (28.6)	16 (35.6)	0.575
Three or more gallstones, *n* (%)	15 (71.4)	27 (60.0)	0.369

LC, laparoscopic cholecystectomy; BMI, body mass index; ASA, American Society of Anesthesiologists; ACC, acute calculus cholecystitis; COPD, chronic obstructive pulmonary disease; WBC, white blood cell; CRP, C-reactive protein.

**Table 4:** Comparison of operative outcomes according to operation time.

Parameter	<104 min (*n* = 21)	≥104 min (*n* = 45)	*P*-value
Operation time (min)	90.0 (90.0–100.0)	130.0 (120.0–160.0)	0.001
Intraoperative blood loss (ml)	20.0 (20.0–20.0)	20.0 (20.0–90.0)	0.086
Bail-out procedure, *n* (%)	1 (4.8)	15 (33.3)	0.012
Open conversion	1 (4.8)	3 (6.7)	1.000
LSC	0 (0)	12 (26.7)	0.007
Surgical complications, *n* (%)			
Wound infection	0	5 (11.1)	0.169
Abdominal collection	1 (4.8)	6 (13.3)	0.416
Choledocholithiasis	1 (4.8)	2 (4.4)	1.000
Bile leak	0 (0)	3 (6.7)	0.546
Adhesive ileus	1 (4.8)	0 (0)	0.318
Postoperative bleeding	0	0	1.000
Bile duct injury	0	0	1.000
Time to oral intake (days)	2.0 (1.0–2.0)	2.0 (1.0–2.0)	0.739
Postoperative hospital stay (days)	6.0 (5.0–8.0)	8.0 (5.0–11.5)	0.051
Total hospital stay (days)	9.0 (7.5–12.5)	10 (8.5–14.5)	0.205
Medicine expenses (CNY)	4,581.8 (2,929.9–6,808.4)	5,473.9 (3,291.6–9,466.7)	0.268
Total expenses (CNY)	26,125.1 (24,003.0–38,836.0)	30,318.0 (27,509.6–43,298.1)	0.158
Mortality, *n* (%)	0	0	1.000

LSC, laparoscopic subtotal cholecystectomy; CNY, China Yuan.

## Discussion

LC is currently the standard surgical modality of ACC management, especially for patients with good medical conditions. Although the early LC for ACC has been well-established by several clinical guidelines, the definition of early LC is still under debate (12). There are currently few studies concerning the feasibility and impact of early LC for mild and moderate ACC irrespective of time since symptoms onset based on the TG18. This study diagnosed and managed ACC of grade I and II according to the TG18 and analyzed their postoperative outcomes. The overall complication rate in this study was 19.7%, while that of Clavien–Dindo grade III morbidity was 12.1%. There was no bile duct injury, postoperative bleeding, and 30-day mortality. There were no statistically significant differences in terms of complication rate, intraoperative blood loss, conversion to bail-out procedures, time to oral intake, postoperative and total hospital stay, medicine expenses, and total expenses between the within and beyond 7 days groups. This suggests the feasibility and safety of early LC for mild and moderate ACC even beyond 7 days since symptoms onset.

Even though many randomized controlled trials (RCTs) have shown that early LC was feasible and safe for ACC, most of these studies were conducted with definitions of early LC as within 72 h since symptoms onset, hospital admission, or patient presentation (13, 14). The meta-analysis that included five RCTs had demonstrated no significant difference in the complication rate or conversion to open surgery between early and delayed LC, while early LC was associated with shorter hospital stay (15). A recent meta-analysis that included 15 RCTs in TG18 found that, compared with delayed LC, early LC for cases within 72 h of patient presentation or symptoms onset was associated with lower cost, hospital stays, mortality rate, complication rate, incidence of bile duct injury, and switching to open surgery (5). Similar results were also obtained with early LC where symptoms’ onset occurred 72 h to 1 week previously (16). Therefore, for ACC patients for whom more than 72 h has passed since symptoms onset, there still are benefits to perform surgery early.

TG07 recommended that early LC for ACC be performed soon after hospital admission, whereas TG13 recommended that early LC be performed soon after admission and within 72 h after symptoms onset (2, 4). The WSES guidelines recommended that early LC for ACC be performed within 7 days from hospital admission and within 10 days from the symptoms onset (3). In TG18, early LC for ACC was proposed regardless of exactly how much time has passed since symptoms onset if a patient is deemed capable of withstanding surgery (5). As it is difficult to determine precisely how many hours have passed since disease onset in the clinical setting, the tendency to neglect the duration of symptoms when proposing early LC for ACC is useful. Although early LC can be performed up to 10 days from symptoms onset, it should be noted that earlier surgery is associated with shorter hospital stay and fewer complications (17). It is worth mentioning that LC for ACC is more complex along with delay of surgery due to severe inflammation, fibrosis, adhesion, and scar change of the gallbladder and its surroundings that progress over time (18). In our study, the median duration of symptoms to LC were 5 and 9 days of within vs. beyond 7 days group, while the operation time was significantly longer and there were more patients with ACC of grade II in the beyond 7 days group. This may be representative of the increased surgical difficulty and cholecystitis severity due to delay of surgery since symptoms onset.

Operation time and conversion to open cholecystectomy are used as indicators of surgical difficulty (9, 19). Advanced age, male gender, severe cholecystitis, higher BMI, elevated CRP or WBC, incarcerated stones in the gallbladder neck, and thick gallbladder wall are few factors that led to prolonged operation time as well as conversion to open surgery (20, 21). Most of patients in this series had one of the above-mentioned criteria, and the operation time was significantly longer in patients in the beyond 7 days group. Further analyses showed that longer operation time was significantly associated with the duration of symptoms to admission, symptoms to LC, and conversion to LSC. As LC may be more difficult to perform in ACC patients with prolonged duration of symptoms, conversion to open cholecystectomy has been recommended to ensure patient safety in difficult LC and to avoid bile duct injury according to TG13. However, LSC is recommended and preferable to open conversion in TG18. In our study, four (6.1%) patients encountered conversion to open cholecystectomy due to severe local inflammation, adhesions, and bleeding from Calot's triangle, which is comparable between the within and beyond 7 days groups. However, more patients underwent LSC in the beyond 7 days group than the within 7 days group [8 (23.5%) vs. 4 (12.5%)], as a CVS cannot be achieved because of nondissectable adhesions, severe fibrosis, and scarring. Based on TG18, we choose conversion to LSC to cope with difficult LC in order to avoid bile duct injury or hemorrhage. Conversion rates are known to range from 3.0% to 30.0% in LC for ACC reported by the literature (22). The low open conversion rate in the current study highlights the efficacy of a bail-out procedure of LSC to resolve difficult LC and avoid direct conversion to open surgery in difficult cases when there is a failure to achieve a CVS.

In ACC, upfront LC becomes more difficult as fibrosis progresses in the inflammatory process when irrespective of the optimal timing of within 72 h since onset. Fibrosis and adhesions surrounding the gallbladder and in Calot's triangle may be severe in ACC (23). As a result of increased difficulty for LC to treat ACC, it is absolutely necessary to avoid any increase in bile duct injury, which is the most dreaded complication of LC in ACC with incidence ranging from 0.2% to 7.0% (24). Based on TG18, we adopted the standardized safe steps in LC to achieve a CVS. The CVS concept was the most commonly recommended option for preventing intraoperative bile duct injury (25). Although current series had three cases (4.5%) of postoperative bile leakage following LSC, there was no bile duct injury. When encountered difficulties to identify local anatomy and achieve a CVS during LC, and then a bail-out procedure of LSC empowered us to successfully finish the difficult surgery. Therefore, the use of standardized safe steps to overcome difficulties, achieving a CVS, and timely conversion to a bail-out procedure of LSC may help minimize bile duct injury.

Subtotal cholecystectomy is recommended in acute settings with frozen Calot's triangle as a rescue method to prevent bile duct injury (9, 12). A recent meta-analysis reported that subtotal cholecystectomy was performed using the laparoscopic (72.9%), open (19.0%), and laparoscopic converted to open (8.0%) techniques (26). Although conversion to LSC led to increased operation time in our study, most LSCs were conducted even 1 h later after failure to achieve a CVS and we attempted to close the remnant in all cases. Three patients emerged with bile leakage after LSC; however, all were managed easily by abdominal drainage. Although postoperative bile leakage was more common following LSC compared with total cholecystectomy, LSC might be an effective surgical technique to avoid biliary injury as reported by other studies (27).

Antimicrobial therapy for patients with Grade I and II ACC is recommended only before and at the time of surgery according to TG18. However, a limitation of the current study was the fact that postoperative antimicrobial therapy was continued until the patient had been ensured without abdominal signs of infection, and the actual duration was about 1–3 days. Furthermore, there were long hospital stays and high wound infection rates in our study. Unfortunately, we took a very conservative postoperative observation for patients in hospital for 3 days. The majority of our study population was elderly with a mean age of 61.8 years and a relatively high comorbidity rate, which slowed down recovery after surgery. In addition, some patients from remote places requested to prolong hospitalization until their wounds were healed. All of which objectively and subjectively led to long hospital stays in our study. Totally 5 wound infection complications occurred in 66 (7.6%) patients, all of which were associated with conversion to bail-out procedures. However, all wound infections were managed easily by wound dressing and local drainage. The high rate of wound infection might be attributed to severe local inflammation and conversion to open cholecystectomy or LSC.

There were other important limitations. It was a single-center retrospective study with a small sample size. Significant biases might have affected the selection of patients for surgery. The relatively small sample size limits the extent of the analyses of risk factors for failure in achieving a CVS as well as conversion to bail-out procedures. Some operative reports were not thoroughly recorded and did not include for analysis, and some key statistics could not be measured. Although the outpatient follow-up was recommended for ACC patient who had underwent surgery, the study still lacked long-term follow-up data to investigate long-term complications. Further prospective investigations should be conducted to confirm the morbidity, mortality, and long-term outcomes of early LC in this group.

In conclusion, our study demonstrated that in a subset of carefully selected patients, applying TG18 in early LC for mild and moderate ACC results in acceptable clinical outcomes. The use of standardized safe steps to overcome difficulties, achieving a CVS, and conversion to a bail-out procedure of LSC to avoid biliary injury in difficult cases are important.

## Data Availability

The original contributions presented in the study are included in the article/Supplementary Material, further inquiries can be directed to the corresponding author.
